# Can we respect the principles of oncologic resection in an emergency surgery to treat colon cancer?

**DOI:** 10.1186/1749-7922-10-5

**Published:** 2015-02-14

**Authors:** Frederico Teixeira, Eduardo Hiroshi Akaishi, Adriano Zuardi Ushinohama, Tiago Cypriano Dutra, Sérgio Dias do Couto Netto, Edivaldo Massazo Utiyama, Celso Oliveira Bernini, Samir Rasslan

**Affiliations:** Division of Clinical Surgery III, Department of Surgery, São Paulo University School of Medicine, São Paulo, Brazil

**Keywords:** Colorectal Cancer, Emergency Surgery, Colorectal emercency surgery

## Abstract

Patients with colorectal cancer admitted to the emergency room are generally at more advanced stage of the disease and are usually submitted to a resection with curative intent in a smaller scale. In such scenario, one of the aspects to be considered is whether the principles of oncologic resection are observed when those patients diagnosed with colon cancer are treated with surgery. We selected 87 patients with adenocarcinoma of colon and/or upper rectum submitted to an emergency surgical resection. The major variables reviewed retrospectively were: the extent of resection performed, the number of dissected regional lymph nodes and the overall survival rate. Intestinal obstruction was observed in 67 patients (77%) while perforation was found in 20 patients (23%). Seven (8%) specimens had circumferential compromised margins, all found in patients with T4 tumors combine with poor clinical status. The number of dissected regional lymph nodes was greater than, or equal to, 12 in 71% of patients. While the average days of stay in the ICU was 5.7 days, the median was 3 days. The morbidity and peri-operative mortality stood at 33.6% and 20%, respectively. The outcome of an emergency surgery of colorectal cancer observed in this study was similar to those found in the literature. The principles of oncologic resection were respected when considering and analyzing the extent of the resection, the surgical margins and the number of dissected lymph nodes.

## Introduction

The Colorectal Cancer (CC) is the third most frequent type of malignancy and the second cause of death by cancer in men and women [1]. It is a common disease in the world, with approximately 850,000 annual new cases and 500,000 deaths annually [2].

According to 2009 statistics from the U.S., 106,100 new cases of colon cancer and 40,870 new cases of rectum cancer are estimated to emerge every year, with about 49,920 deaths in the U.S. [3]. In Brazil, according to the National Cancer Institute (INC) numbers from 2009, 12,490 new annual cases of CC were likely to be found in men and 14,500 new cases in women. The statiscs show an estimated risk of 13 new cases in every 100,000 men and 15 per 100,000 women [4].

Despite preventive measures and early detection, from 6% to 30% of patients have symptoms or late complications related to the disease, requiring an emergency intervention [5–7].

Patients with colorectal cancer admitted to an emergency room are at a more advanced stage of the disease and are submitted to resection with curative intent in a smaller scale. A bowel obstruction also increases the risk of perforation and is accompanied by elevated rates of potential local recurrence.

Colorectal cancer surgical intervention performed to address an acute obstruction is associated with a mortality of 15% to 20% and a morbidity of 40% to 50%, which are significantly higher than in an elective situation [8–12]. These elevated rates of morbidity and mortality are generally related to apparence of the CC in its more complicated forms: obstruction, intestinal necrosis, perforation, diffuse or localized peritonitis; also aggravated by others diseases which some of these patients already have.

Considering such circumstances, one of the aspects to be considered is whether the principles of oncologic resection are observed when patients with colon cancer are subjected to an emergency surgical treatment.

## Objectives

This study aims to examine whether the principles of cancer surgery could be observed in the operations performed in patients who had been admitted in the emergency room, with a previous diagnosis of CC, with the presence of intestinal obstruction or perforation. The extent of resection, the microscopic analysis of resected surgical margins and number of regional lymph nodes were observed in this study.

As a secondary objective, the analysis aims to describe the demographic characteristics of the population observed, complications related to surgical treatment and the long-term survival of those patients.

The study selected 87 patients from all people admitted in the Emergency Room Service. The persons who were chosen had been diagnosed with adenocarcinoma of colon and/or upper rectum and were submitted to a surgical resection.

Patients with a diagnosis other than the adenocarcinoma of the colon, primary tumor in the middle or lower rectum, or not submitted to colectomy were excluded.

We have retrospectively reviewed data on age, race, sex, presence of comorbidities and status classification of risk factors according to the criteria set by the American Society of Anesthesiologists (ASA) (Table 1).

**Table 1:** **Demographics and risk factor stratification of the population**

Average age (years)	60 (24–89)
**Race (white/all)**	**71/87**
**Systemic hypertension**	**34/87**
**Diabetes mellitus**	**6/87**
**Chronic pulmonary obstructive disease**	**1/87**
**ASA**	
**I**	**39/87 (45%)**
**II**	**38/87 (44%)**
**III**	**8/87 (9%)**
**IV**	**2/87 (2%)**

Certain variables specifically related to the malignancy under analysis, which have been considered were: complications of the cancer disease in the clinical presentation (obstruction or perforation), site of primary tumor in the large intestine, staging with the TNM system of the American Joint Committee on Cancer (AJCC), and histological type, according to its degree of cellular differentiation.

Certain variables related to the colectomy were also analyzed and they were: the extent of resection performed, the number of dissected regional lymph nodes and the final outcome of the resection, considering the presence or absence of residual disease. The study also examined the percentage of patients who underwent primary anastomosis versus those who underwent resection with stoma procedures.

Postoperative complications were divided into local and systemic. The number of days of hospitalization in the ICU and the overall survival rate were also observed.

## Results

Review of medical record data (Table 2) revealed a predominance of sigmoid and right colon cancer. Intestinal obstruction was observed in 67 patients (77%) while perforation was found in 20 patients (23%). Bleeding was never observed in these cases.

**Table 2:** **Site of the primary tumor according to the colonic anatomic division**

Localization	N	%
**Right colon**	**37**	**42,6**
**Transverse colon**	**5**	**5,8**
**Left colon**	**14**	**16**
**Sigmoid**	**27**	**31**
**Upper rectum**	**4**	**4,6**

Primary anastomosis was performed in 48 patients (55%), while the remainder (45%) resections were performed with stoma procedures.

The extent of the resections performed, the number of primary anastomotic dehiscence and the anatomical site of the colon resection are shown in Table 3. In the right colon, 89% patients were submitted to primary anastomosis and 12% developed anastomotic dehiscence. In the left colon, primary anastomosis was performed in 27% of the cases and no dehiscence was observed. In the sigmoid colon and upper rectum, only five primary anastomosis were performed (16.6%) and no further complications were observed.

**Table 3:** **Extent of colon resection performed, number of primary anastomosis and number of dehiscences in the population studied**

Right colectomy	Anastomosis	33	Dehiscence	4 (12%)
	Colostomy	4		
Left colectomy	Anastomosis	3	Dehiscence	0
	Colostomy	8		
Sigmoidectomy	Anastomosis	5	Dehiscence	0
	Colostomy	25		
Transversectomy	Anastomosis	2	Dehiscence	1 (50%)
	Colostomy	2		
Total colectomy	Anastomosis	5	Dehiscence	0

Seven (8%) specimens of surgical resection had circumferential compromised margins. Those patients had bulky T4 tumors - four with tumors in the upper rectum and three with tumors located in the right colon. Four of these patients had bowel perforation presented as a primary symptom. Multivisceral *en bloc* resection was precluded due to poor clinical status of the patients.

Histological analysis of the degree of cellular differentiation of the resected specimens revealed well differentiated tumors in 63 patients (72%), moderately differentiated adenocarcinoma in 15 (17%) and poorly differentiated adenocarcinoma in nine of the patients (11%).

The number of regional lymph nodes which were dissected was greater than or equal to 12 in 71% of patients undergoing resection of the tumor, with a mean of 4.02 metastatic lymph nodes (median of 1).

**Figure 1:**
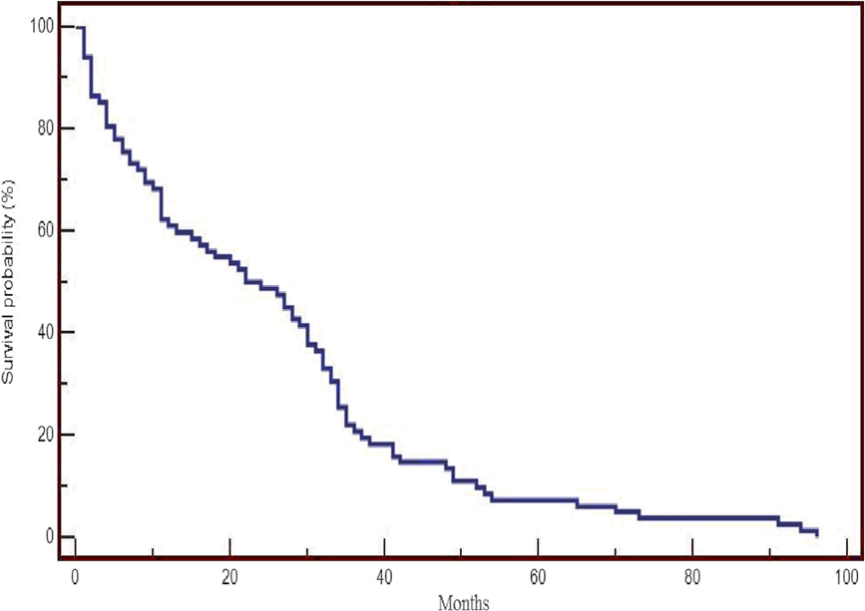
**Overall Survival on Kaplan Meyer curve.**

Table 4 shows the rates of major postoperative complications in the group of selected patients. There was dehiscence in 10.4% of the anastomosis, evisceration in 2.2% of patients and other infectious complications, such as septic shock (5.7% of the patients) and infected wound (6.8%). The rates of overall survival, according to pathological staging at three and five years, are shown in Tables 5 and 6 and the Kaplan-Meyer curve (probability of survival) is demonstrated in Figure 1.

**Table 4:** **Number and percentage of local and systemic complications of the population submitted to the emergency operation**

Complications	n	%
Anastomotic dehiscence	5	10,4
Evisceration	2	2,2
Infected wound	6	6,8
Septic shock	5	5,7
Pneumonia	3	5,7
Colostomy complications	2	5,1

**Table 5:** **Number of patients operated on emergency in accordance with anatomical and pathological TNM staging of AJCC/UICC 6th edition**

Stage	N	%
I	1	1,1
IIa	11	12,7
IIb	4	4,6
IIIa	4	4,6
IIIb	17	19,6
IIIc	12	13,8
IV	38	43,6

**Table 6:** **Overall survival at three and five years according to AJCC/UICC 6** ^**th**^ **ed**

Stage	Overall survival at three years	Overall survival at five years
I	**100,0%**	**0%**
II	**50,0%**	**21%**
III	**9%**	**9%**
IV	**10%**	**10%**

The average days of stay in the ICU was 5.7 days, with a median of three days, while the morbidity and peri-operative mortality (30 days after surgery) were 33.6% and 20%, respectively.

## Discussion

A review of the literature shows colon cancer patients admitted to the emergence room present more frequently a bowel obstruction (8% to 60% of cases) [13, 14], followed by perforation (2% to 22% of cases) [15–17] and bleeding. In our study, 77% of patients had intestinal obstruction while the remainder presented a perforation. Almost half (42.6%) of our patients had right colon cancer and 36% of patients had sigmoid or upper rectum cancer.

The emergency operation is usually associated with higher rates of morbidity, mortality and worse prognosis when compared to an elective surgery. Both overall survival rate and percentage of survival specifically related to cancer are lower in patients with CC operated on an emergency situation. When compared with elective surgery patients, those diagnosed with obstructive colorectal cancer submitted to an emergency surgery have a two-fold higher risk of dying as a result of their disease, despite the attempt of a curative operation [18]. Smothers et al., in 2003, published the first case–control study of patients who were operated on because of an emergency related to colorectal cancer (Table 7). The results showed an emergency colectomy as an independent negative prognostic factor in terms of morbidity and mortality related to the surgery. In their series, the authors have found a morbidity and mortality related to emergency operation of 64% and 34%, respectively [19]. Tobaruela et al., (Table 7) in a review of 51 patients operated on for obstruction or perforation, found a morbidity and mortality of 41% and 15% respectively. The overall survival was 15% in 62 months [20]. Ascanelli et al., in 2003, found 27% of morbidity and 12% of mortality in 118 patients submitted to emergency surgery [21].

**Table 7:** **Morbidity and mortality: Literature analysis**

Authors	Series	Morbidity	Peroperative mortality
Tobaruela et al. [20]	51	41%	14%
Smothers et al. [19]	29	64%	34%
Ascanelli et al. [21]	118	27,1%	11,9%
Current Series	87	33,6%	20%

In our series of 87 patients, we also observed increased rates of morbidity and mortality: 33.6% and 20% respectively (Table 7).

A peculiarity in this study was the higher-than-reported rate of dehiscence in right colectomy (12%) [22]. The right colectomy with primary anastomosis in an emergency intervention is considered to have lower rate of anastomotic complications, although Candelária et al. have also shown significant rate of anastomotic dehiscence in his series [23]. Another relevant aspect in the treatment of CC in an emergency, a primary objective of this study, concerns the feasibility of the appropriate oncologic resection with curative intent. Oncologic principles have been established in accordance with practical parameters which include: (1) extent of resection and negative surgical margins, (2) en bloc resection of contiguous tissue attached to the primary tumor, (3) lymph node dissection of at least 12 regional lymph nodes examined by pathologists [24, 25]. It is unclear if the oncologic principles of surgery can be, in fact, implemented in the scenario of emergency operations, although it can be seen a small number of publications demonstrating the efforts to follow these principles in patients with a complicated form of colon cancer [12–30]. Recently, it was suggested that the principles of oncologic resection for CC operated on an emergency can be met, also achieving results related to the long-term survival [31]. Some clinical and surgical aspects are considered when choosing an oncologic resection in the emergency setting: the fear of causing further physiological deterioration in a critical patient; the possibility of extending the resection and the time spent in surgery; the difficulty of making an appropriate lymph node dissection; the struggle to manipulate and mobilize the distended colon; and the potential severe contamination and inflammation of the peritoneal cavity in cases involving bowel perforation are factors adversely affecting the choice of an oncologic resection.

It has been suggested a surgery for CC performed by specialized surgeon has a significant impact on survival [32]. Surgeons who attend the emergency setting have variable degrees of specialization. However, the majority of them are less specialized and they may perform a resection intended only to address the urgency of the situation without respecting oncologic principles.

The first criterion considered in our study concerns the extent of resection and the pathological status of the surgical margins. In general, clear margins can be obtained even in an emergency situation. With about 5 cm to 10 cm of margins, the epiploic and paracolics lymph nodes can be removed and the risk of anastomotic recurrence minimized. In the group studied, 92% of patients had R0 resection. Although the achievement of clear margins is technically simple for colectomies, the study observed 8% of positive microscopic margins, all in T4 tumors, which could be related to the impossibility of *en bloc* multivisceral resection, since these patients might have presented poor clinical conditions. The second criterion examined was the number of dissected regional lymph nodes, which has prognostic and therapeutic implications. The resection of all metastatic lymph nodes also defines an R0 resection and a sample of at least 12 lymph nodes is needed to secure appropriate accuracy. In this series, 71% of patients had at least 12 regional lymph nodes dissected. The absolute number of lymph nodes dissected may be influenced by the extent of the resection and was higher in patients undergoing total or subtotal colectomy.

## Conclusion

The study shows the outcome of an emergency surgery of colorectal cancer was similar to those found in the literature. It was possible to respect the principles of oncologic resection, as regards the extent of resection, surgical margins and lymph node dissection. The morbidity and mortality were higher, however, the different rates were attributed to further complications of the disease and the clinical condition of some of the patients than to the fact those patients had undergone a left colectomy and sigmoidectomy. In the group of patients submitted to right colectomy, we have observed a higher rate of dehiscence of the ileo-transverse anastomosis, superior to the percentage reported in the literature. It has been possible to respect the oncologic principles of resection in the emergency surgery for colorectal cancer.
